# Early and late selection: effects of load, dilution and salience

**DOI:** 10.3389/fpsyg.2014.00248

**Published:** 2014-03-20

**Authors:** Tal Makovski, Bernhard Hommel, Glyn Humphreys

**Affiliations:** ^1^Department of Psychology, The Open University of IsraelRaanana, Israel; ^2^Cognitive Psychology Unit, Leiden UniversityLeiden, Netherlands; ^3^Department of Experimental Psychology, Oxford UniversityOxford, UK

**Keywords:** attention, early selection, late selection, perceptual load, dilution, salience

The present issue focuses on the classic question of early vs. late selection and evaluates the current status of the perceptual load theory that has been offered as an intermediate solution. Several of the papers compare or contrast perceptual load with an alternative explanation- perceptual dilution. The first group of papers report evidence that is inconsistent with the perceptual load theory but is generally consistent with the dilution theory.

Roper and Vecera (2013) report that flanker effects can be found under high perceptual load. Extending the duration of the display, and particularly of the relevant target, produces reliable congruency effects even under high perceptual load. The authors therefore argue that factors such as stimulus and encoding demands contribute to the load effect and that visual short term memory serves as an additional bottleneck when stimuli are briefly presented. Yeshurun and Marciano's (2013) findings also challenge the perceptual load theory. The authors found that task difficulty, as manipulated by degradation of visual information, did not affect attentional selection and flanker interference. This is in contrast to the claim that increasing sensory load increases distractor interference. Furthermore, the basic load effect was not replicated in all 4 experiments, and flanker effects were found even under high perceptual load.

Mevorach et al. (2014) tested patients with unilateral neglect and found that contralesional neutral elements eliminated the interference presented by a distractor. The authors argue that given the notion that no attentional resources are allocated to the contralesional field, perceptual load should not be affected by presenting items in the contralesional field. Instead they suggest that neutral stimuli dilute the flanker effect and that attentional selection is determined by dilution rather than load. This is in line with Benoni and Tsal (2013) who present a critical review of perceptual load theory. They challenge the theory's assumptions and supporting evidence, and provide supportive arguments for the alternative dilution theory.

Chen and Cave (2013) further studied the dilution effect. They present data that is consistent with dilution but not with perceptual load. However, they argue that the current conception of dilution is simplified. In particular the processing of neutral items is not only dependent on the number of stimuli present but also on complex interactions between top-down and bottom-up processes. Thus, both distractor and neutral elements in a multi element display compete for the same limited attentional resources.

In their opinion paper Linnell and Caparos (2013) argue that in accord with the perceptual load theory the spatial profile of attention was more focused when perceptual load was high and less focused when it was low. However in contrast to the theory this holds only when cognitive resources were available. Indeed, the authors emphasize the role of cognitive engagement in the task at hand and suggest that variations in perceptual load modulate task difficulty and this in turn alters cognitive engagement and motivation, factors often neglected in the study of attention.

More evidence for a strategic component in the seemingly automatic processing of task-irrelevant information comes from Biggs and Gibson (2013). They show that prior experience and situational expectations modulate the degree to which irrelevant information is processed. As they argue, this might render the assumption of a broad versus narrow allocation of visual attention in explaining effects of irrelevant information processing superfluous.

The review of Scalf et al. (2013) presents a hybrid neural competition theory that is generally consistent with both perceptual load and dilution theories. This theory reinforces the original view that low perceptual load is associated with a stronger impact of task-irrelevant information. As the authors point out, this might reflect different processing strategies in conditions with high and low perceptual load: While low perceptual load might allow for bottom-up-driven target selection, high perceptual load might call for top-down regulation. The latter leads to stronger filtering, which reduces the impact of task-irrelevant distractors.

The remaining papers use perceptual load theory as a direct or indirect context for studying other aspects of attentional selection. The role of working memory in regulating the degree to which distractors can be ignored is the focus of de Fockert's (2013) review. In support of the original assumption, the review provides strong evidence that higher working memory load makes it more difficult to ignore task-irrelevant distractors. This fits with the idea that working memory has an active role in gating irrelevant information.

Forster (2013) takes the perceptual load theory a step forward into the realm of mind-wandering and thought distraction. In her review she carefully distinguishes between different types of task relevancy and between external and internal (e.g., task-unrelated thoughts) sources of distraction. She argues that perceptual load theory is a powerful and largely universal framework to study distraction effects.

Parks et al. (2013) used SSVEPs together with ERPS to study the effect of attentional load in a go-no task. The findings reveal a center-surround configuration of both facilitation and suppression in the visual field.

Swallow and Jiang (2013) bring in a novel perspective by relating findings on the impact of perceptual load to the attentional boost effect—the observation that distractor processing can benefit from temporal synchronicity with target presentation. As they point out, the seemingly automatic processing of distractors with high perceptual load might reflect a kind of “intentional automatization”: the cognitive system might be programmed to take in information automatically whenever being triggered by a target, suggesting that automatic processing might be a byproduct of intentional selection.

Folk (2013) makes an interesting conceptual distinction between processing costs produced by response-incompatible distractors on the one hand and search costs on the other. By combining aspects of the original perceptual-load paradigm and the classical singleton-search paradigm, he provides evidence that search costs remain even under conditions where the compatibility of distractors no longer affects processing.

Finally, Moher et al. (2013) tested selection processes without the explicit requirement of target identification. They found that detection performance remained high in spite of focal attention manipulations (i.e., target saliency, availability of cognitive resources, and familiarity) that eliminated identity-repetition effects. Thus, the authors conclude simple target detection is not dependent on focal attention.

We hope you will find this Research Topic interesting and informative. Enjoy your reading!
